# Income inequality and mass shootings in the United States

**DOI:** 10.1186/s12889-019-7490-x

**Published:** 2019-09-20

**Authors:** Roy Kwon, Joseph F. Cabrera

**Affiliations:** Department of Sociology, University of La Verne, 1950 Third Street, La Verne, CA 91750 USA

**Keywords:** Mass shootings, Income inequality, Socioeconomic factors

## Abstract

**Background:**

Mass shootings are an increasingly common phenomenon in the United States. However, there is little research on whether the recent growth of income inequality is associated with this rise of mass shootings. We thus build on our prior research to explore the connection between income inequality and mass shootings across counties in the United States.

**Methods:**

We assemble a panel dataset of 3144 counties during the years 1990 to 2015. Socioeconomic data are extracted from the U.S. Bureau of the Census. Mass shootings data are from three databases that compile its information from the Federal Bureau of Investigation and media sources, respectively. These data are analyzed using random effects negative binomial regressions, while controlling for seven additional predictors of crime.

**Results:**

Counties experiencing a one standard deviation growth of income inequality witnessed 0.43 more mass shootings when using the definition of three or more victim injuries (incidence rate ratio [IRR] = 1.43; 95% confidence interval [CI] = 1.24, 1.66; *P* < .001) and 0.57 more mass shootings when using the designation of four or more victim deaths (IRR = 1.57; 95% CI = 1.26, 1.96; *P* < .001).

**Conclusions:**

Counties with growing levels of income inequality are more likely to experience mass shootings. We assert that one possibility for this finding is that income inequality fosters an environment of anger and resentment that ultimately leads to violence.

## Background

From a movie theatre in Aurora, Colorado, to an elementary school in Newtown, Connecticut, it seems mass shootings are becoming a more common occurrence in the United States. This claim is supported by the data as the number of mass shootings involving three or more victim-related injuries increased from a total of 8 in the 1970s, to 115 in the current decade by the end of 2015 [1]. What is particularly alarming about this trend is that the impact of mass shootings typically extends well beyond the immediately targeted areas, as the media attention and public scrutiny generated by mass shootings tend to produce fear, hysteria, and moral panics at the city-, state-, and even national-level [2]. In short, mass shootings produce both an immediate human toll as well as widespread societal repercussions.

Similarly, one of the more noteworthy changes in developed economies over the past few decades is the dramatic growth of income inequality. According to the United Nations [3], the increasing bifurcation of national income is particularly acute in the United States, where the top 0.1%’s income increased by 4.0% annually between 1980 to 2011, while the bottom 99%’s income increased by only 0.6% annually from 1976 to 2007. Noteworthy in this regard is many scholars find income inequality is linked to a number of social problems, such as increased crime and homicide rates [4–6]. However, there are no studies to date examining whether this increase of inequality is connected to mass shootings. An important question thus remains unanswered in the empirical literature: is the contemporary growth of income inequality associated with the recent rise of mass shootings in the United States?

Numerous sociologists and criminologists over the years explored the correlates of overall homicide rates at the population-level. This research provides a good starting-point for understanding how income inequality may contribute to mass shootings [4–6]. These researchers largely draw on a relative deprivation perspective to explain the connection between economic disparities and violence. According to Robert Merton [7], an early forerunner of this perspective, communities with large differences of household income maintain an environment of anger, frustration, resentment, and hostility. Referred to as goal blockage, the effects of relative deprivation are particularly severe when a population finds it difficult to achieve socioeconomic success and status [8].

To this end, research in public health and epidemiology provides some concrete evidence that income inequality can produce an unstable and hostile social environment. According to Wilkinson and Pickett [9], inequality is strongly associated with feelings of status insecurity, which is an important predictor of stress and anxiety. Researchers also show that those exposed to environments with a higher probability of being judged negatively by others, which should be more common in unequal environments, tend to possess greater levels of stress and other negative health outcomes [10, 11].

Furthermore, research from psychology shows that social inequality is not only associated with stress and anxiety, but also aggression. According to this literature, people exposed to unequal environments are more likely to internalize the social norms of power and domination, as opposed to equality and reciprocity. Specifically, those socialized in unequal environments are skeptical of notions of justice and fairness, which promotes hostility and violence [12, 13]. Similarly, others suggest that the salience of competition as typically found in unequal environments may lead to violence and homicide [14], while related research points to a potential relationship between inequality and the prevalence of youth bullying [15, 16].

Finally, the findings of a recent experimental study further supports the logic of the relative deprivation perspective [17]. In this research, scholars examined whether exposing economy passengers to first class passengers affected the rate of air rage. The results demonstrated that economy passengers who walked through the first-class cabin were more than twice as likely to experience an air rage incident vis-à-vis economy passengers who did not walk through the first-class cabin. Although this study is regarding situational inequality, it shows how even a brief encounter with an unequal environment may lead to aggressive behavior. In light of these observations, we test whether the recent *growth* of income inequality is associated with mass shootings at the population-level. We do so by building on our prior research [18], where we find that differences between counties in their *level* of income inequality is linked to mass shootings.

## Methods

We used panel regression techniques where mass shootings over a 10 year-period are regressed on the first-difference of independent variables measured during the years 1990 to 2000 and 2000 to 2010, respectively (e.g., mass shooting _2000 to 2009_ = inequality_2000–1990_ +controls_2000–1990_). The data are thus composed of county-decade observations.

### Dependent variables

The dependent variable is the total count of mass shootings from the Mass Shootings in America (MSA) dataset (https://library.stanford.edu/projects/mass-shootings-america). These data are from media reported accounts of mass shootings. Consistent with the literature, the MSA excludes shootings that are identifiably gang- or drug-related. The definition of mass shootings is a source of debate. Disagreements revolve around the minimum number of victim injuries and/or deaths that qualify as a mass shooting. Definitions thus vary, with some using the broader three or more victim injuries threshold [1], while others use the restrictive cutoff of four or more victim deaths [19, 20]. We prefer the broader definition of three or more injuries as it allows for more variability in the dependent variable, i.e., more mass shooting incidents. However, to ensure our results are not an artifact of a particular definition, we also retest all models using the more restrictive threshold of four or more deaths.

We replicate our findings using two additional data sources: Mother Jones (http://www.motherjones.com/politics/2012/12/massshootings-mother-jones-full-data) and USA Today (https://www.usatoday.com/story/news/nation/2013/09/16/mass-killings-data-map/2820423/). Mother Jones is similar to MSA in that the mass shootings information is collected from media sources, while USA Today data are compiled using the FBI’s (Federal Bureau of Investigation) supplementary homicide report which is only then supplemented with media sources. Although the USA Today dataset is useful given its dissimilar source of information, a disadvantage is that these data are reported starting only in 2006, limiting the temporal scope of the analysis based on this dataset. As a final note, both alternative datasets only use the more restrictive mass shootings definition of four or more deaths. For a more detailed discussion of mass shootings measurement strategies and critiques, see Huff-Corzine et al. [21].

### Independent variables

All independent variables are from the U.S. Bureau of the Census [22–24]. All covariates are drawn from the crime and homicide literature [4–6]. The main independent variable in this study is income inequality. This covariate is represented by the post-tax version of the Gini coefficient, which is a measure that varies between 0 and 100, with higher scores denoting greater levels of income inequality. Income inequality is widely used in the predictors of homicide literature to capture the concept of relative deprivation. In addition, all models are estimated net of poverty rates, which is the percent of households earning below the federal poverty line. This variable is used in literature to serve as a proxy for absolute deprivation. We include poverty in the models as it allows us to ensure that the link between income inequality and mass shootings is due to *relative differences* of income and not the result of *resource scarcity*.

There are seven additional control variables. Unemployment rate is the percent of the population over the age of 16 that is without work and actively seeking employment. Scholars note that the social problems associated with unemployment are a major predictor of homicide. Population density is the number of individuals residing in a given county per square mile. This captures the argument that elevated levels of population density produce anomie and social disorganization, resulting in higher crime and delinquency rates. Young population are those aged between 15 to 29 as a percent of the population and accounts for the view that adolescents and younger adults are more likely to engage in crime. Minority population is measured as a percent of the population and controls for the higher rate of violence in minority communities. High school graduation rate is the percent of the population that is above the age of 25 with at least a high school or equivalent degree. The final two variables are popular measures of gun control laws: 1) right to carry legislation and 2) assault weapons bans [19, 25, 26]. These are state-level dummy predictors (no = 0, yes = 1) and are only included in the multilevel models (MLM).

### Data analysis

The analysis uses STATA 13.0 to examine the IRR of mass shootings using random effects negative binomial regression with robust clustered standard errors, which is regularly used with rare-events dependent variables. All independent variables are logged to reduce positive skew and z-score standardized to allow for the direct comparability of the IRR. Furthermore, to ensure that our results are replicable when using different definitions or data sources of mass shootings, we also report three robustness checks. First, although we favor the broader mass shootings definition of three or more injuries, we retest our regression models using the more restrictive threshold of four or more fatalities. Second, in addition to using the MSA data, we also present alternative models in which the regressions are re-estimated using the mass shootings data from Mother Jones and USA Today. And finally, we present multilevel model (MLM) results to ensure that our findings are not an artifact of a particular statistical technique. In these models, observations or time are nested in counties, which are in turn nested in states. An additional benefit of MLMs is that they allow us to control for state-level gun control legislation.

It is important to note that we performed a number of pre- and post-regression diagnostics to ensure we are using the most optimal technique for the data in question. A summary of all pre- and post-regression tests, correlation matrix, and additional robustness checks, are available upon request from the corresponding author.

## Results

**Fig. 1:**
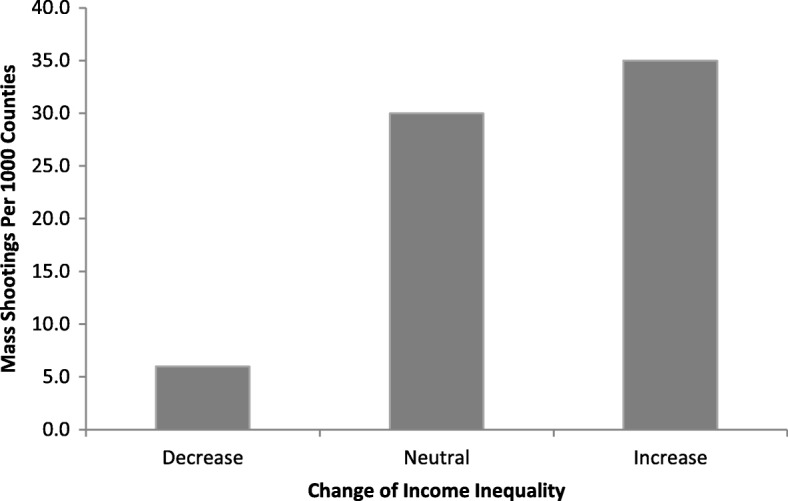
County-Level Mass Shooting Rates by Change in Income Inequality

Figure 1 provides information on mass shootings. These data are presented by grouping counties together by their level of change in income inequality. According to the data, during each time-period examined, counties experiencing a decrease of income inequality witnessed a mass shootings rate of 6 per 1000 counties, counties with a negligible change of income inequality experienced 30 per 1000, and counties with an increase of income inequality observed 35 per 1000. These data are useful as they provide preliminary descriptive evidence that recent inequality dynamics may be associated with mass shooting patterns at the county-level.

The results of the negative binomial regressions are available in Table 1. These equations regress the number of mass shooting events on the first difference of income inequality, net of the first difference of the control variables. Starting with the main results using the MSA data, the findings indicate that regardless of whether a mass shooting is defined as three or more injuries (IRR = 1.43; CI = 1.24, 1.66; *P* < .001) or four or more deaths (IRR = 1.57; CI = 1.26, 1.96; *P* < .001), income inequality produces a significant positive association. This means counties that experience a one standard deviation increase of inequality will observe approximately 0.43 to 0.57 more mass shootings. In contrast, poverty rate fails to produce significant results when using the MSA data for both the three or more injuries (IRR = 1.35; CI = 0.98, 1.87; *P* = .06) and four or more deaths (IRR = 0.97; CI = 0.64, 1.47; *P* = .89) definition. Furthermore, these results remain similar when we replicate the findings using different mass shootings data sources. That is, income inequality continues to return a significant positive association when using the Mother Jones (IRR = 1.43; CI = 1.12, 1.82; *P* < .01) and USA Today (IRR = 1.26; CI = 1.04, 1.52; *P* < .05) data, while poverty remains a non-significant predictor across both Mother Jones (IRR = 0.83; CI = 0.47, 1.47; *P* = .53) and USA Today (IRR = 1.23; CI = 0.64, 2.34; *P* = .52).

**Table 1:** Incidence Rate Ratios of Mass Shootings in U.S. Counties

	MSA: Three or More Injuries	MSA: Four or More Deaths	Mother Jones: Four or More Deaths	USA Today: Four or More Deaths
	Adj. IRR (95% CI)	Adj. IRR (95% CI)	Adj. IRR (95% CI)	Adj. IRR (95% CI)
Income inequality	1.43 (1.24, 1.66)	1.57 (1.26, 1.96)	1.43 (1.12, 1.82)	1.26 (1.04, 1.52)
Poverty rate	1.35 (0.98, 1.87)	0.97 (0.64, 1.47)	0.83 (0.47, 1.47)	1.23 (0.64, 2.34)
Unemployment rate	1.20 (1.02, 1.41)	1.15 (0.92, 1.44)	1.21 (0.92, 1.60)	1.05 (0.78, 1.41)
Population density	0.27 (0.08, 0.88)	0.22 (0.08, 0.57)	0.18 (0.06, 0.55)	0.39 (0.02, 7.39)
Young population	1.07 (0.84, 1.37)	0.99 (0.70, 1.39)	1.21 (0.89, 1.66)	1.09 (0.86, 1.39)
Minority population	6.27 (1.79, 21.99)	14.36 (5.51, 37.39)	18.01 (7.00, 46.36)	3.53 (0.68, 18.35)
HS graduation rate	0.59 (0.44, 0.78)	0.59 (0.39, 0.90)	0.57 (0.33, 0.97)	0.53 (0.36, 0.78)
2010–2015	2.81 (1.99, 3.98)	1.58 (0.98, 2.55)	1.38 (0.79, 2.42)	1.24 (0.78, 1.95)
Wald Chi-Square	92.96	56.86	50.11	24.99
AIC	1363.44	737.53	545.40	817.02
BIC	1430.88	804.97	612.84	884.46
County-Decades (N)	6273	6273	6273	6273

*Adj. IRR* adjusted incidence rate ratio, *CI* confidence interval. All variables are logged and z-score standardized. Robust standard errors clustered by county reported

We also report a set of MLM models in Table 2 that replicate the previous results. The value of this approach is that it allows us to retest our findings by controlling for state-level gun control legislation: right to carry laws and assault rifle bans. Consistent with the main results, the MLM models show that income inequality remains a significant positive predictor of mass shootings when testing the MSA’s version of the three or more injuries (IRR = 1.56; CI = 1.33, 1.83; *P* < .001) and four deaths (IRR = 1.62; CI = 1.34, 1.96; *P* < .001) designations. Also similar to previous findings is that poverty continues to return non-significant results across both the three or more injuries (IRR = 1.28; CI = 0.89, 1.83; *P* = .17) and four or more deaths (IRR = 0.98; CI = 0.61, 1.57; *P* = .94) definition. Robustness checks using alternative data sources return corroborating evidence, as inequality is a significant positive predictor when using Mother Jones (IRR = 1.46; CI = 1.09, 1.97; *P* < .05) and USA Today (IRR = 1.32; CI = 1.04, 1.69; *P* < .05), while poverty fails to generate significant results for both the Mother Jones (IRR = 0.81; CI = 0.48, 1.38; *P* = .45) and USA Today (IRR = 1.29; CI = 0.66, 2.53; *P* = .44) datasets.

**Table 2:** Incidence Rate Ratios of Mass Shootings in U.S. Counties, Multilevel Models

	MSA: Three or More Injuries	MSA: Four or More Deaths	Mother Jones: Four or More Deaths	USA Today: Four or More Deaths
	Adj. IRR (95% CI)	Adj. IRR (95% CI)	Adj. IRR (95% CI)	Adj. IRR (95% CI)
Level 1 = County-Level				
Income inequality	1.56 (1.33, 1.83)	1.62 (1.34, 1.96)	1.46 (1.09, 1.97)	1.32 (1.04, 1.69)
Poverty rate	1.28 (0.89, 1.83)	0.98 (0.61, 1.57)	0.81 (0.48, 1.38)	1.29 (0.66, 2.53)
Unemployment rate	1.15 (0.88, 1.50)	1.09 (0.80, 1.48)	1.17 (0.80, 1.71)	0.95 (0.63, 1.42)
Population density	0.13 (0.00, 1.87)	0.16 (0.29, 0.93)	0.14 (0.02, 0.92)	0.60 (0.00, 48.61)
Young population	1.01 (0.77, 1.34)	0.97 (0.68, 1.37)	1.19 (0.86, 1.63)	1.07 (0.81, 1.40)
Minority population	4.59 (0.70, 29.95)	16.31 (3.92, 67.87)	21.03 (4.84, 91.33)	3.08 (0.33, 28.09)
HS graduation rate	0.66 (0.47, 0.92)	0.65 (0.42, 1.01)	0.66 (0.40, 1.08)	0.65 (0.39, 1.07)
2010–2015	2.98 (1.97, 4.51)	1.78 (1.07, 2.99)	1.51 (0.77, 2.96)	1.35 (0.75, 2.43)
Level 2 = State-Level				
Right to carry laws	0.63 (0.19, 2.06)	6e^−30^ (6e^− 125^, 5e^+ 65^)	0.18 (0.00, 34.43)	0.08 (0.00, 24.13)
Assault rifle ban	0.88 (0.25, 3.09)	1.33 (0.45, 3.89)	1.37 (0.41, 4.49)	1.72 (0.60, 4.94)
Wald Chi-Square	127.77	61.65	40.98	16.01
County-Decades (N)	6273	6273	6273	6273

*Adj. IRR* adjusted incidence rate ratio, *CI* confidence interval. All variables are logged and z-score standardized. Robust standard errors clustered by county reported

## Discussion

There is strong evidence in this study to suggest the recent growth of income inequality is significantly associated with mass shootings in the United States. Specifically, this evidence indicates that a one standard deviation increase in the growth of income inequality augments the number of mass shootings by 0.43 to 0.57. In contrast, there is no evidence that poverty rates are associated with these events. We end below by discussing both the contributions and limitations of the current study.

The earliest mass shootings research was performed almost exclusively by psychologists and mental health professionals using interviews of a few individuals [27, 28]. Since these earlier studies, scholars started analyzing a wider array of cases to develop typologies of mass murder [29, 30], while others focused on the demographic characteristics of victims and shooters [31, 32]. Crucial is that this focus on the individual-level predictors of mass shootings serves as the basic foundation of the current public policy discourse, which focuses heavily on how mental illness is connected to mass shootings. However, there is reason to be skeptical of this proposed causal connection, especially since less than 5% of all firearm killings are attributable to those with mental illness, a proportion that translates to a rate that is lower than the national average for those without a mental illness [33].

Surprisingly, it was not until recently that researchers started exploring the population-level predictors of mass shootings using states as the unit of analysis [19, 25, 26]. However, while two of these three studies include demographic variables in their regressions (e.g., poverty, unemployment, population, young population, etc), none of the covariates analyzed are consistently significant. Furthermore, these researchers focus on the implications of gun control legislation, and none of these works analyze the effect of income inequality. Most recently, although some scholars are starting to examine how different levels of income inequality may be connected to mass shootings using counties as the preferred unit [18, 34], there is no research on how the recent growth or change of income inequality is associated with this phenomenon. This study thus adds to the mass shootings literature and public policy debate by shifting the focus away from popular individual-level explanations and towards understudied population-level factors.

A number of interesting findings deserve some additional discussion. To begin with, the results indicate that various policy strategies designed to reduce gun violence are not significantly associated with mass shootings, which is consistent with previous research [19, 25]. Rather, the clear conclusion from our results is that socioeconomic factors, such as income inequality, are the main driver of mass shootings in the United States. In light of this observation and the widely recognized role of income inequality for other social problems [9, 16], it may be prudent for academics and policymakers to identify policies that increase general social welfare in order to solve the mass shootings epidemic in the United States.

In addition, although inconsistently significant across the various models tested, a number of control variables produce significant results and are signed in a counterintuitive direction: population density, minority population, and high school graduation rate. With population density, other studies find that the level of population density is significantly associated with mass shootings [18, 34]. As such, our finding that change in this variable decreases mass shootings may indicate there is a upper threshold at which continued increase in population density no longer increases mass shootings. In terms of minority population, our discovery that an increase in minority population is positively associated with mass shootings is consistent with previous findings [35]. According to this research, an increase in the minority population results in ethnic fractionalization in the United States, resulting in decreased civic engagement and social integration, thus leading to more violence and mass shootings. And finally, that the high school graduation rate increases mass shootings may be explained by the so-called Kuznets Inverted-U hypothesis [36, 37]. According to this line of reasoning, as the educated population increases, there is more potential for the bifurcation of the labor force into higher- and lower-paid occupations, resulting in higher levels of income inequality. As such, the positive association between education and mass shootings may, at least in part, reflect the potential contribution of education to income inequality dynamics.

There are some shortcomings in the current study that need to be addressed. An issue with the MSA dataset is that all sources are from media reports. Since the internet was not available at the beginning of this dataset, earlier mass shootings may be underreported. Furthermore, obscure incidents are less likely to be included as they tend to draw less media attention. These concerns are addressed in two ways. First, we re-estimate all regression models by decade in Tables 3 and 4, with results showing that the connection between income inequality and mass shootings is relatively stable over time. In fact, the connection becomes more robust during later years, when the dataset should be least biased. Second, we also replicated our results using the USA Today dataset in the previously discussed Tables 1 and 2, which gathers its information mainly from the FBI. These checks provide some assurance that the link between inequality and mass shootings is not an artifact of shortcomings in the data.

**Table 3:** Negative Binomial Models: Incidence Rate Ratios of Mass Shootings in U.S. Counties

	MSA: Three or More Injuries		MSA: Four or More Deaths	
	MS_2000–2009_ = ∆ Covariates_1990–2000_	MS_2010–2015_ = ∆ Covariates_2000–2010_	MS_2000–2009_ = ∆ Covariates_1990–2000_	MS_2010–2015_ = ∆ Covariates_2000–2010_
	Adj. IRR (95% CI)	Adj. IRR (95% CI)	Adj. IRR (95% CI)	Adj. IRR (95% CI)
Income inequality	1.40 (1.05, 1.88)	1.42 (1.17, 1.72)	1.54 (1.12, 2.11)	1.56 (1.12, 2.15)
Poverty rate	0.82 (0.48, 1.38)	2.10 (1.24, 3.55)	0.66 (0.43, 1.00)	1.87 (0.86, 4.08)
Unemployment rate	0.85 (0.60, 1.22)	1.10 (0.88, 1.37)	0.94 (0.66, 1.34)	0.99 (0.67, 1.47)
Population density	0.31 (0.09, 1.05)	0.05 (0.00, 0.57)	0.24 (0.08, 0.67)	0.72 (0.00, 0.93)
Young population	0.75 (0.53, 1.07)	3.10 (2.16, 4.44)	0.83 (0.54, 1.28)	2.68 (1.65, 4.35)
Minority population	1.28 (0.12, 13.15)	7.15 (3.07, 16.64)	6.40 (1.01, 40.50)	11.76 (3.78, 36.58)
HS graduation rate	0.38 (0.21, 0.70)	0.76 (0.51, 1.14)	0.33 (0.21, 0.54)	0.93 (0.65, 1.33)
Wald Chi-Square	46.99	92.70	45.33	69.17
County-Decades (N)	3134	3139	3134	3139

*Adj. IRR* adjusted incidence rate ratio, *CI* confidence interval. All variables are logged and z-score standardized

**Table 4:** Multilevel Models: Incidence Rate Ratios of Mass Shootings in U.S. Counties

	MSA: Three or More Injuries		MSA: Four or More Deaths	
	MS_2000–2009_ = ∆ Covariates_1990–2000_	MS_2010–2015_ = ∆ Covariates_2000–2010_	MS_2000–2009_ = ∆ Covariates_1990–2000_	MS_2010–2015_ = ∆ Covariates_2000–2010_
	Adj. IRR (95% CI)	Adj. IRR (95% CI)	Adj. IRR (95% CI)	Adj. IRR (95% CI)
Level 1 = County-Level				
Income inequality	1.47 (1.06, 2.02)	1.50 (1.14, 1.97)	1.54 (1.12, 2.11)	1.63 (1.15, 2.33)
Poverty rate	0.76 (0.39, 1.47)	2.46 (1.50, 4.03)	0.59 (0.39, 0.90)	2.50 (1.06, 5.91)
Unemployment rate	0.83 (0.57, 1.22)	1.24 (0.90, 1.69)	0.96 (0.58, 1.59)	0.94 (0.49, 1.78)
Population density	0.26 (0.03, 2.05)	0.00 (0.00, 0.16)	0.16 (0.01, 1.71)	0.04 (0.00, 3.50)
Young population	0.74 (0.48, 1.13)	2.98 (1.65, 5.39)	0.83 (0.51, 1.34)	2.54 (1.36, 4.75)
Minority population	0.87 (0.07, 9.85)	6.58 (2.08, 20.80)	8.61 (1.07, 69.35)	10.43 (2.81, 38.67)
HS graduation rate	0.19 (0.06, 0.59)	0.99 (0.85, 1.16)	0.19 (0.03, 0.96)	1.00 (0.85, 1.18)
Level 2 = State-Level				
Right to carry laws	4e^−32^ (3e^−65^, 50.74)	1.05 (0.39, 2.86)	1e^−36^ (9e^−40^, 3e^−33^)	0.40 (0.00, 48.93)
Assault rifle ban	1e^−29^ (3e^− 32^, 3e^−27^)	0.84 (0.19, 3.64)	0.16 (0.00, 1972.26)	2.27 (0.95, 5.37)
Wald Chi-Square	20.17	86.93	22.48	63.22
County-Decades (N)	3134	3139	3134	3139

*Adj. IRR* adjusted incidence rate ratio, *CI* confidence interval. All variables are logged and z-score standardized

Another limitation of our dataset is the result of a change in the census’s data collection procedure. Previously, although both the long and short forms of the census were administered every 10 years, the long form was replaced with the American Community Survey (ACS) in the year 2010. This change is important since the long form contains smaller margins of error than the ACS. Yet when we retest our models by decade in Tables 3 and 4, the results for income inequality remain positive and stable across the various time periods. This suggests that the census’s alternation of their collection methodology does not play a major role in our results.

And finally, some may observe that the removal of gang-related shootings from the dataset may limit the findings since income inequality is associated with gang-related violence [38]. The datasets used in the current study do not allow for the inclusion of these types of shootings. However, there are two reasons this is not a concern for our study. First, given the aforementioned research, it is likely the inclusion of gang-related violence will improve the connection between inequality and mass shootings. And second, the current research is less concerned with estimating the predictors of traditional street crime (e.g., gang-related turf wars), the predictors of which may potentially be different from mass shootings.

## Conclusions

This study provides evidence that counties with growing levels of income inequality experience more mass shootings. In addition, scholars show that today’s more pressing social problems are highly correlated with inequality [9]. Given the evidence, the major policy implication of our study is that part of the solution to solve the growing mass shootings epidemic, and a litany of other social problems, may involve creating policies that can reduce the growing income inequality between Americans.

## Data Availability

The data sources used for the current study are publically available through the websites noted below. 1). U.S. Bureau of the Census: http://www.census.gov/data.html 2). Mass Shootings in America: https://library.stanford.edu/projects/mass-shootings-america 3). Mother Jones: http://www.motherjones.com/politics/2012/12/massshootings-mother-jones-full-data 4). USA Today: https://www.usatoday.com/story/news/nation/2013/09/16/mass-killings-data-map/2820423/

## Abbreviations

ACS: American Community Survey
CI: Confidence interval
FBI: Federal Bureau of Investigation
IRR: Incidence rate ratios
MLM: Multilevel model
MSA: Mass Shootings in America Database
